# Adolescent Voices in Action—Co-Designing Digital Sexual and Reproductive Health Knowledge Translation Interventions: Community-Based Participatory Action Project

**DOI:** 10.2196/71148

**Published:** 2025-12-01

**Authors:** Salima Meherali, Amyna Ismail Rehmani, Mariam Ahmad

**Affiliations:** 1 Faculty of Nursing College of Health Sciences University of Alberta Edmonton, AB Canada

**Keywords:** co-design, digital interventions, sexual and reproductive health, youth-friendly, knowledge translation

## Abstract

**Background:**

Adolescents need comprehensive education and resources to promote their sexual and reproductive health (SRH) and make informed decisions about their SRH. However, many adolescents fail to secure an opportunity to learn accurate and reliable SRH information, as they face sociocultural barriers, fear of prejudice, and societal stigma. Information available on digital platforms may not always be evidence-based and can further spread misinformation. Digital knowledge translation interventions that provide evidence-based SRH information in North America are limited.

**Objective:**

This study aimed to co-design a digital knowledge translation intervention, reflective of adolescents’ voices, to provide evidence-based, accessible, and accurate SRH resources.

**Methods:**

We conducted a large multisite project across 3 cities in Canada. Using a community-based participatory approach and principles of human-centered design, we established adolescent advisory groups (AAGs) to actively engage them in the design, development, and implementation of the intervention.

**Results:**

A total of 26 participants were recruited from Edmonton, Vancouver, and Toronto to be part of the advisory groups. AAG members participated in design-thinking sessions to brainstorm ideas for website design, identify informational needs for content development, provide iterative feedback on the design of the intervention, and suggest strategies to improve engagement and interaction. With their input, 6 priority areas were identified to develop SRH resources (ie, puberty, menstruation, sexually transmitted infections, healthy relationships, sexual assault, and contraception). Their feedback informed the design’s language, visual appeal, and engagement factors. To promote meaningful engagement of AAGs at each step, we used strategies such as gamification activities, group discussions, and flexible scheduling, resulting in high retention and ownership of the process among AAG members.

**Conclusions:**

Co-designing with adolescents strengthened the intervention’s cultural relevance, youth-friendliness, and credibility. Our process emphasizes the significance of involving adolescents in co-designing SRH interventions, which results in more meaningful, long-term, and youth-friendly solutions.

## Introduction

Adolescence is an essential stage of growth and development that sets the foundation for all the consecutive stages of life [1]. For adolescents to grow healthy and develop successfully, they require access to information about their health and well-being, including comprehensive sexual and reproductive health (SRH) education [2]. As adolescents begin to explore their identities and experience significant changes (eg, puberty), SRH education becomes fundamental to protect them from negative SRH consequences, such as unintended pregnancy and sexually transmitted infections (STIs), and equip them to improve their sexual health and well-being (eg, healthy relationships) [3,4]. However, adolescents do not always have the opportunity to access reliable and accurate SRH information and often face barriers, such as stigmatization, discrimination, provider prejudice, and embarrassment while seeking help or accessing SRH resources [5,6]. These barriers are deeply rooted in sociocultural norms that stigmatize healthy sexuality and SRH, particularly among females; lesbian, gay, bisexual, transgender, queer or questioning, and plus (others; LGBTQ+) youth; and those from immigrant communities [7,8].

A significant challenge that these adolescents encounter is the lack of reliable and trustworthy sexual health information and services, which can be attributed to restrictive policies, caregiver’s or provider’s unwillingness to openly discuss sexual health issues, parental control, and lack of confidentiality [5,6,9]. Such challenges are intensified for adolescents from migrant backgrounds as they struggle with balancing cultural, religious, and familial expectations pertaining to their SRH, systemic issues (eg, language barriers and lack of culturally safe resources), and navigating access to SRH services dedicated to immigrant and marginalized populations [7,10]. Previous research with immigrant adolescents in Canada has documented how they often encounter structural barriers within health and education systems, which limit their access to SRH services and lead them to rely on informal digital sources (eg, Google and YouTube [Google]), which may not be evidence-based and can promote misinformation [4,8].

Digital knowledge translation (KT) interventions have the potential to offer promising and equitable venues for influencing various health practices while addressing barriers, including stigma, confidentiality concerns, and difficulties in receiving support [5,9,11,12]. KT interventions facilitate the dissemination of research-based information to health care consumers in a user-friendly format to meet information needs, including but not limited to mobile apps, websites, multimedia content (videos, infographics, etc), and social media platforms [13]. Particularly, in the realm of SRH, digital KT interventions have the potential to reach vulnerable and marginalized populations while providing anonymity and privacy on sensitive SRH matters [5,6,14]. A systematic review on the effectiveness of digital sexual health interventions reported that a higher proportion of website-based interventions were found to improve sexual health outcomes in adolescents, which was demonstrated by an increase in knowledge and access to sexual health services, improvement in STI prevention practices, and reduction in unhealthy sexual behaviors [15]. We conducted an environmental scan to understand the range of mobile health apps dedicated to adolescent SRH in Canada and found that very few apps offered comprehensive, reliable, and evidence-based information [16]. Furthermore, the findings combined with other literature in the field also highlighted gaps in cultural adaptations within such interventions and the need for youth-centered design in SRH digital intervention development [7,16,17].

Co-design is a participatory action research approach that brings together community members, stakeholders, and researchers to collaboratively develop, design, and implement interventions to address issues within the community [17,18]. This approach aims to actively and meaningfully engage individuals in the research process to understand their needs and integrate their experiences to design innovative solutions and improve outcomes [19,20]. This is particularly significant when working with adolescents, as it allows researchers to gain insights into their unique and creative perspectives and determine what would work best for them. Engaging adolescents as equal partners in the design process empowers them to take ownership and responsibility for the process, promoting the success of an intervention. The World Health Organization’s framework for planning, developing, and implementing youth-centered digital solutions recommends that for digital interventions to succeed and be sustainable, adolescents should be meaningfully engaged at each step of the planning, design, and implementation process [2]. This ensures that the intervention is adolescent-friendly and accessible, acceptable, equitable, appropriate, and effective among youth [2]. The co-design approach is uniquely positioned to bridge gaps for immigrant adolescents by incorporating cultural perspectives and SRH information needs, addressing issues of trust and accessibility, and ensuring that the style and content of information delivery resonate with their lived realities [17].

Co-designed health initiatives are gaining traction for influencing health behaviors and improving health outcomes [12,17,21]. Several studies have documented the benefits of digital KT interventions in promoting health and well-being among adolescents, particularly with SRH outcomes [5,7,22,23]. Regardless, digital interventions that provide comprehensive, evidence-based, and reliable SRH education for adolescents, especially from migrant backgrounds, are somewhat limited in North America [16]. Also, a few studies have explored co-design in the context of immigrant adolescents’ SRH, especially within Canada. Therefore, our aim was to co-design adolescent-friendly digital SRH KT interventions with and for immigrant adolescents in Canada to provide easy access to resources and services at their convenience with anonymity. In this study, we outline the process of engaging immigrant adolescents in the co-design of an evidence-based website as a KT intervention aimed at promoting SRH information.

## Methods

### Study Design

**Figure 1:**
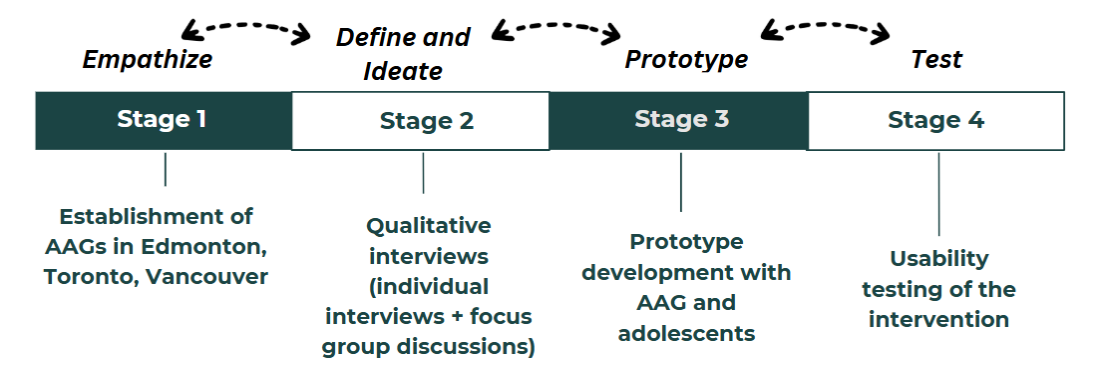
Stages of the project. AAG: adolescent advisory group.

We conducted a larger multisite project (2023-2024), intending to co-design digital SRH interventions for immigrant adolescents in Canada. Conducting a multisite project helped to ensure that the intervention would reach immigrant adolescents across Canada. We integrated the principles of community-based participatory action research and human-centered design (HCD) to maximize end user engagement at each step of the co-design process, which guaranteed that the outcome would be effective and sustainable [17,24]. HCD, which is also referred to as design thinking, is a creative and systematic problem-solving approach that prioritizes the needs, desires, and experiences of individuals at the core [24]. It not only focuses on the functionality of a certain solution but also considers the user’s motivation, behavior, and challenges, ensuring that it is culturally relevant [24]. Building on these principles, we divided our project into 4 stages (Figure 1). This process helped us to keep adolescents’ perspectives and needs at the center of the co-design process and maximize collaboration, participation, and engagement [18].

### Stage 1: Establishment of Adolescent Advisory Groups

We first developed and convened adolescent advisory groups (AAGs) in Edmonton, Toronto, and Vancouver regions. These cities have one of the highest percentages of immigrant populations in Canada [25]. Our purpose was to develop a mutual partnership with immigrant adolescents to actively involve them in various research activities, such as data collection, data analysis, resource development, concurrent feedback, and knowledge mobilization.

### Research Team Composition

The research team, associated with recruitment and training of AAGs and coordinating co-designing activities, included the principal investigator (PI; SM), the project coordinator (MA), and a research assistant (AIR). The PI who conceived this project has extensive experience with community-based research methodologies and adolescent SRH initiatives with a desire to promote equitable access to SRH resources and services, especially among marginalized populations. We partnered with the Department of Computing Science at the University of Alberta to seek professional support and expertise in co-designing digital resources. The Vice Dean, along with graduate research assistants, dedicated their skills and knowledge to develop the website prototype, incorporate user feedback and preferences, and provide technical guidance at each step of the project. The research assistant (AIR) and project coordinator (MA) were responsible for developing content, connecting with SRH experts for content validation, organizing AAG meetings and activities, coordinating with the team in the Department of Computing Science to track progress, and completing research coordination tasks. With this mutual collaboration, we were able to design our digital intervention effectively and consult on technological-related issues.

### Setting and Participants

Through multifaceted strategies, we recruited adolescents aged 12-19 years from various cultural and ethnic backgrounds, ages, and genders, and invited them to participate in our AAG meetings. We included adolescents who were either born outside of Canada and immigrated to Canada (first-generation immigrants) or who were born in Canada but had at least 1 foreign-born parent (second-generation immigrants). Our inclusion criteria required that participants reside in Canada at the time of the study, be able to communicate in English, and have access to a device with internet access to connect with the research team online. Research assistants, in collaboration with various youth organizations, community networks, and adolescent-friendly clinics, recruited adolescents to be part of the AAGs. We also promoted recruitment posters through social media advertising (eg, Facebook [Meta] and Instagram [Meta]) to target our population. We made efforts to develop recruitment materials and strategies to be inclusive of LGBTQ+ youth and those living with disabilities. After screening and recruitment of potential participants, the included AAG members were given a comprehensive explanation of the study objectives, its intended outcomes, risks and benefits, and implications. They were provided with a detailed information sheet, highlighting the particulars about the study and their participation expectations.

The AAGs were established based on practical considerations (eg, the number of adolescents interested in participating) and as per the recommendations of research team members, who had extensive experience working with AAGs. We decided that each advisory group at each site should consist of a reasonable target size of 8-12 adolescents to result in meaningful engagement (expected total AAG participants: 24-30). This sample size enabled us to exercise deeper relationship-building, promoted active participation, and gave opportunity to each member to express their voice. Literature on participatory research design encourages smaller group sizes to maximize closer collaboration, equitable participation, and maintain a balance between diverse perspectives and group dynamics [17,24]. AAG members in each city were expected to meet online on Zoom (Zoom Communications) throughout the project and share their insights through design-think sessions on the development of the website and SRH resources through a systematic process.

### Procedures (Co-Design Process)

To foster meaningful engagement, AAG members were provided with initial training on SRH concepts, qualitative and quantitative research methods (types of research methods, data collection, and interview methods), social determinants of health, and the process of adolescent engagement. An important component of the training sessions was to evaluate the effectiveness of the training, which we assessed through various strategies. For example, we asked AAG members to develop some sample open-ended interview questions to demonstrate their understanding of ethical research practices and their comprehension of research concepts. Each site AAG meeting or session was held separately and was scheduled based on members’ availabilities. The sessions started in February 2023. After their training and familiarization with the project, the AAG members met regularly once every month, starting in July 2023 until January 2024 for approximately 60-90 minutes to discuss project updates, recruitment activities, and planning of further activities. As we developed the prototype and started an iterative cycle of feedback and integrating changes, the meetings were organized every other month until August 2024. All the meetings were conducted online over Zoom and were audio-recorded and transcribed verbatim after identifying information was removed. Furthermore, the research assistant and project coordinator maintained detailed notes and meeting summaries to document the co-design process and AAG feedback to capture their input in real time and ensure that their feedback was accurately reflected at each step.

The meetings were moderated by the PI (SM), project coordinator (MA), and research assistant (AIR). The moderators prompted active participation from all members through various techniques (eg, blackboard activities, quizzes, and feedback forms), to ensure that each participant was able to share their thoughts, feelings, and experiences in a safe space. Given the sensitive nature of SRH topics, we ensured to apply a trauma-informed and youth-centered facilitation approach. All team members were trained to identify any signs of discomfort and distress, and a distress protocol was developed to guide facilitators in responding to signs, including pausing the discussion and offering follow-up support. Participants were encouraged to join from private spaces and were reminded of their right to skip any questions, take breaks, or keep their cameras off. Recognizing the potential for power imbalances between researchers and AAG members, we implemented measures to enhance equitable participation, such as using youth-friendly language, giving options for providing feedback, ensuring all feedback was equally valued, and regularly consulting members on meeting structure, preference, and decision-making.

All members tried to attend all meetings, considering their academic commitments and other scheduling conflicts, but all members remained part of the advisory group throughout the project. Despite the occasional absences, the members were proactive in following up with the research team and identified ways to contribute their insights either during sessions or through asynchronous feedback. Throughout the AAG meetings, we facilitated healthy discussions about the learning and informational needs of adolescents, the cultural or social influences on SRH education, and the quality of the SRH education curriculum offered in schools.

### Data Analysis

We used a descriptive qualitative approach to synthesize feedback and identify recurrent ideas related to SRH information needs, engagement strategies, and co-design experiences. Meeting transcripts, facilitator logs, and session summaries were reviewed by our research team to identify recurring topics, key insights, and decisions made collaboratively with youth. Two researchers (AIR, MA) independently reviewed the session notes to ensure consistency in interpretation and enhance analytic trustworthiness through iterative discussion and consensus. We kept a detailed log of all AAG interactions and systematically organized the information into categories to support the decision-making process. After evaluating the findings from these sessions, as well as the qualitative interviews conducted in stage 2 (findings are published elsewhere), we finalized priority areas to develop SRH educational content.

### Ethical Considerations

We received ethics approval for this project from the University of Alberta Research Ethics Board (August 26, 2021; Pro00113664), the Toronto Metropolitan University Research Ethics Board (April 27, 2022; REB 2022-069), and the University of British Columbia (May 31, 2022; #H22-00395). We followed the Tri-Council Policy Ethical Conduct for Research Involving Humans [26] principles for seeking consent, which does not specify an age of consent for children. Following these guidelines, we determined whether adolescents had the capacity to understand the objectives, significance, implications, risks, and benefits of the study by explaining these topics and asking them to reiterate based on their understanding. The ethics committee from all 3 boards approved and stipulated that if participants were capable enough to understand the procedures and study details, they could provide consent for themselves. After addressing any questions, participants were asked to sign a consent form to confirm their voluntary participation as AAG members, along with a confidentiality agreement to protect the identities of fellow members and the experiences shared within sessions. All participants were deemed capable of providing informed consent for themselves. Participants were given an honorarium of CAD $20 (approximately US $14) per hour for participating in various activities.

## Results

### Participant Characteristics

We recruited a total of 26 participants for this stage of the study, with 88.4% (n=23) of participants identifying as female and 11.5% (n=3) as male. Out of the 26 participants, 53.8% (n=14) of participants were first-generation immigrants and 46.1% (n=12) were second-generation immigrants. Almost half of the participants were 19 years of age, whereas only 1 participant was 15 years old. None of the participants formally withdrew or disengaged from the project. The demographic information of participants is presented in Table 1.

**Table 1:** Demographic characteristics of adolescent advisory group (n=26).

Characteristics				Participants, n (%)		
**Sex**						
	Female			23 (88.4)		
	Male			3 (11.5)		
**Age (y)**						
	15			1 (3.8)		
	16			2 (7.6)		
	17			3 (11.5)		
	18			7 (26.9)		
	19			13 (50)		
**Place of birth**						
	Northern America			12 (46.1)		
	Sub-Saharan Africa			5 (19.2)		
	Asia			7 (26.9)		
	Europe			1 (3.8)		
	South America and Caribbean			1 (3.8)		
**Languages spoken at home (multiple responses)**						
	English			25 (96.1)		
	Urdu			4 (15.3)		
	Hindi			3 (11.5)		
	Cantonese			1 (3.8)		
	Farsi			1 (3.8)		
	Vietnamese			1 (3.8)		
	Others (Tamil, Dari, Bengali, Gujarati, Somalian, and Tagalog)			6 (23)		
**Number of years in Canada**						
	1-3			2 (7.6)		
	4-9			6 (23)		
	10 or more			6 (23)		
	Lived in Canada all or most of life			12 (46.1)		
**Current level of education**						
	Grade 7-10			1 (3.8)		
	Grade 11			2 (7.6)		
	Grade 12			3 (11.5)		
	Postsecondary school			20 (76.9)		
**Place of birth of parents**						
	**Mother**					
		Northern America	1 (3.8)			
		Sub-Saharan Africa	10 (38.4)			
		Asia	13 (50)			
		Europe	1 (3.8)			
		South America and Caribbean	1 (3.8)			
	**Father**					
		Sub-Saharan Africa	10 (38.4)			
		Asia	14 (53.8)			
		Europe	1 (3.8)			
		South America and Caribbean	1 (3.8)			

### Design Process

To initiate the development of SRH digital resources offered through our website, we consulted AAG members, stakeholders, and the interdisciplinary research team iteratively to co-develop the best course of action for planning, designing, and implementation. We started with brainstorming potential names for our resource website and its initial layout and design. After discussing many acronyms, we collectively decided to name our website PEER, which stands for Partnership to Empower adolescent Engagement in Research. This not only emphasizes the role that adolescents play in promoting research initiatives but also signifies our work to empower adolescents to drive changes by forming partnerships. We also reflected on various themes for our website and which color would be the most appealing to adolescents. Although some participants shared that bright colors (eg, dark blue and yellow) would be captivating, others commented that these colors might only complement some text and graphics. Therefore, we collectively decided on a neutral color tone (ie, light purple) with a white background. Our development team designed some sample logos and design patterns for the website, which were shared with AAG members.

### Development and Delivery of Content

We finalized 6 priority areas to focus our SRH content on: puberty, menstruation, STIs, sexual assault, contraception, and healthy relationships. Our team developed evidence-based resources for every topic. We ensured all topics were covered at length and that common misconceptions regarding these topics were addressed. We identified the information to be delivered within each topic with the help of AAG members. We wanted the content to be informative and engaging at the same time. Hence, we did not rely solely on text-based information but also developed infographics, animations, and videos along with textual information to keep users engaged. We collaborated with 6 stakeholders and experts in the field (SRH researchers, clinicians, sexual health consultants, and SRH or public health nurses) to validate the content and ensure that it is factual, evidence-based, and unbiased. With their feedback, we were able to improve the language and delivery of the content as well as rectify any errors. For instance, validators suggested using language that does not blame an individual and is inclusive and gender-neutral (eg, menstruating people instead of menstruating girls).

**Figure 2:**
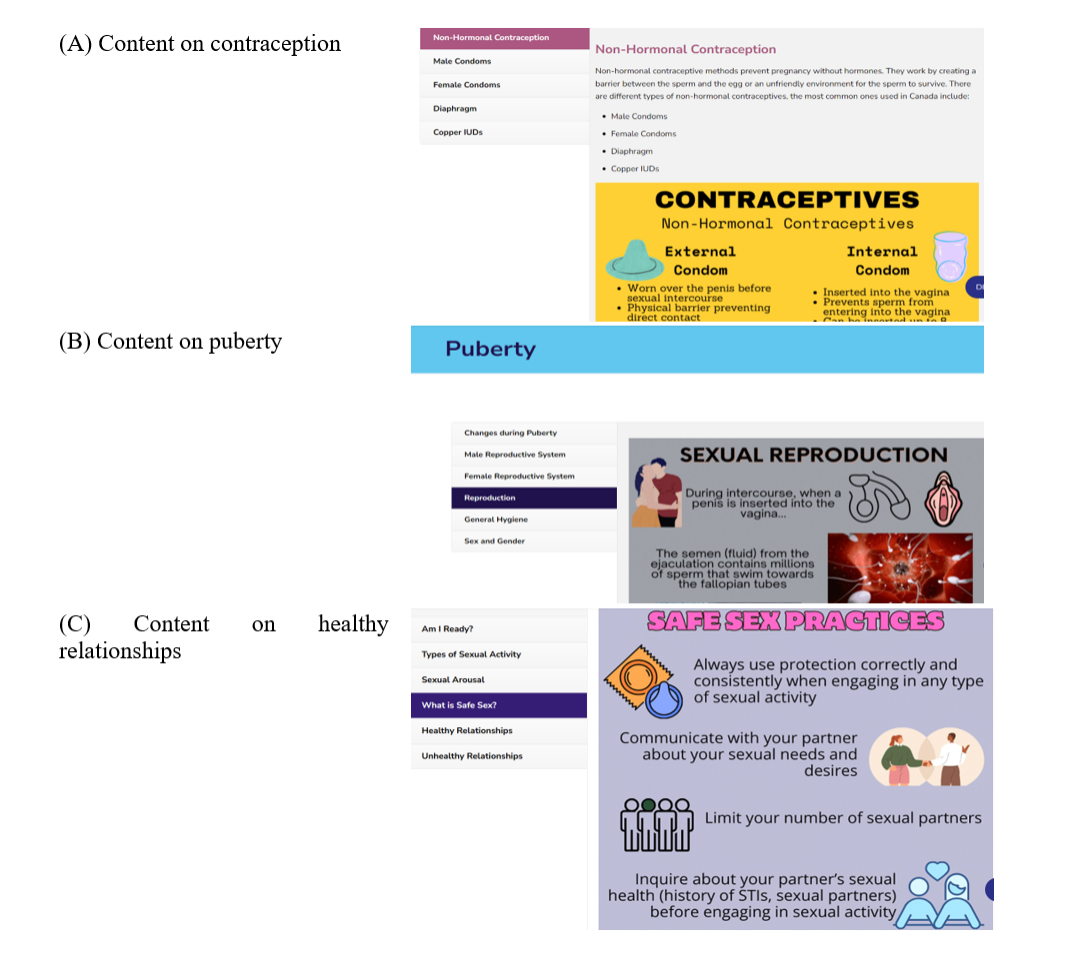
Examples of youth-centered sexual and reproductive health content developed for the Partnership to Empower Adolescent Engagement in Research platform.

Following content validation, we shared the SRH resources with our AAG members (sample content in Figure 2). They appreciated the simple language used to explain complex issues and the different formats that we used to deliver information. They also provided valuable feedback to improve our resources. We initially developed our content videos with audio voice-over using computer software, but members found the voice to be unnatural and robotic. They suggested that the research team record the voice-over instead to give it a more human touch. We welcomed their feedback, and a member of our team did the voiceover. In addition, members re-emphasized that the text under each category should be concise, in bullet form, and have graphics throughout. We considered their suggestions and tried to keep the information concise and bite-sized. Regarding the display of content on the website, we initially organized the subtopics (headings) through horizontal widgets. However, many AAG members did not find this format appealing and suggested that the widgets be organized vertically instead.

**Figure 3:**
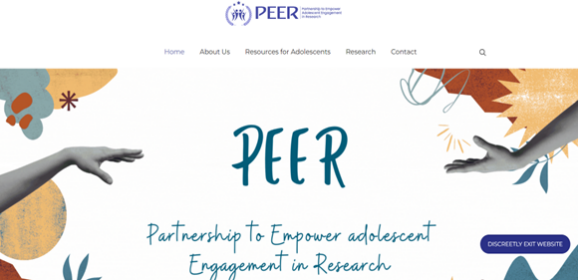
Landing page of the Partnership to Empower Adolescent Engagement in Research digital platform.

Integrating all the feedback from the AAG and stakeholders, we finalized the first prototype of our website. We agreed to display a video instead of a still image on the landing page as it seemed more attractive and engaging to the users (Figure 3). Also, we made the edits suggested by the AAG members within the SRH resources and rearranged some tabs on the website. As per their suggestions, we finalized the display and organization of the content. A sample of these resources is presented in Figure 2.

We implemented various strategies to collect comprehensive feedback from AAGs on the prototype of the website and SRH resources. Some participants shared detailed feedback through Zoom sessions, whereas others also agreed to provide written feedback through Google Forms. We organized the feedback received from the members and collectively decided whether to implement the change or not. Some features were specifically difficult to implement at this stage as they required advanced technological adaptations (eg, helper chat). Table 2 illustrates some examples of feedback from our members and what steps were taken to address the suggestions.

**Table 2:** Feedback and response.

Prototype feedback	Team decision
“I would like for the bottom tab to be cleaner with the Land Acknowledgement and the Social links. YouTube is the only icon on a new level and the Land Acknowledgement left aligned text with the title on the left is a bit of putting. Maybe adding this acknowledgement on the About Us page makes more sense.”	All the icons at the bottom of the page were aligned on the same line and adjusted to provide a better interface.
“I think the infographics should follow a color theme and be cohesive in one section. The headings could be bolded to make them stand out. All the infographics can be centered in the middle of the webpage.”	Headings were bolded and graphics centered on the page. Fonts kept consistent across topics.
“Maybe a helper chat or who could answer any questions.”	Feature cannot be added at this point.
“I think adding some basic information about some common reproductive issues/disorders such as PCOS or endometriosis would also help adolescents because some girls get irregular periods, which becomes scary.”	Topic of menstruation expanded to include content on common menstrual disorders (eg, PCOS).
“To make the website better, I would maybe move the search bar upwards to somewhere easier for the viewer to see, instead of having to scroll down. That way in every section of the site you can search what you’re looking for.”	Search bar added to the top right corner as well.
“Change the color of the font. The font of the subtext on the website is very light gray and doesn’t stand out as much. It is a little hard on the eyes to read.”	All the text font change to black color.
“Include an option to quickly exit the website if learning SRH content and need to close the tab immediately (exist fast button).”	“Discretely exit website” button added to redirect user to a different web page.
“Make it more ‘adolescent friendly’ by adding brighter color schemes, more stimulating buttons, and more enticing for teens to want to be interested in.”	More colors added with brighter graphics and fonts; each individual tab with a different brighter color.

## Discussion

### Principal Findings

Our project aimed to co-design adolescent-friendly digital SRH resources by actively engaging AAG. This process provided valuable insights into best practices for adolescent participation in digital health intervention development. The formation of our AAGs follows best practices for meaningful youth engagement, as recommended in the literature [17-20,27]. We ensured that meaningful youth engagement was not only fundamental but deeply ingrained in our methodology. By empowering adolescents to actively participate in the design and execution of our intervention, we pushed beyond tokenism and manipulation, which can hinder genuine engagement [17,27]. Instead, adolescents were involved as co-creators, with their ideas, input, and leadership influencing every stage of creation.

We recruited a diverse group of adolescents aged 12-19 years from various cultural and ethnic backgrounds through schools, community organizations, and social media platforms. This approach represented different perspectives across age groups, cultures, and gender identities [28,29]. To prepare AAG members for effective contributions, we conducted workshops introducing key research concepts and SRH topics. This training addressed a gap in previous studies where adolescent advisory members often lack formal preparation [30,31]. Armed with this foundational knowledge, AAG members were then engaged in the co-design process using various participatory techniques. Throughout the AAG meetings, we used engagement methods tailored to different age groups, including gamified activities for younger adolescents and in-depth discussions for older teens. We also used structured feedback forms and open discussions. This diverse approach is supported by research showing that creative engagement methods can enhance youth involvement and input [24]. The iterative nature of our process, with regular meetings and opportunities for feedback, allowed for continuous refinement of the intervention based on adolescent input, a fundamental principle of co-design [32].

Our approach incorporated several effective strategies from previous studies in website development. We involved adolescents in naming the intervention, choosing color schemes, and determining content formats, aligning with recommendations for youth-centered design in digital health interventions [33]. Prototypes and iterative feedback cycles mirrored successful co-design processes described in other digital health projects targeting youth [12,20,21,34]. For example, the study by Wright et al [23] highlights how youth advisors in their study broadened the scope of their proposed digital intervention through an iterative co-design approach and helped to bring forward an innovative strategy to improve information-seeking behaviors. Our experience highlighted the importance of flexibility when working with adolescents. Offering online participation options and accommodating schedules enhanced engagement, consistent with findings from other youth advisory group studies [35]. In addition, providing compensation for AAG members’ time and contributions promoted long-term engagement and valued their input.

AAG members played a crucial role in evaluating content readability and suggesting more adolescent-friendly language, addressing the need for accessible digital health resources. Their involvement in discussions about privacy features and discreet access options aligned with research emphasizing the importance of confidentiality in adolescent-oriented sexual health interventions [36]. For instance, AAG members suggested implementing a “quick exit” feature and disguising the website icon, which was incorporated into the final design to enhance user privacy. During pilot testing, users highly praised these features, with many expressing that these privacy measures made them feel more comfortable using these resources. The AAG members also expressed satisfaction with how their suggestions were implemented, reinforcing the value of their input in the design process.

In addition to the significance of the co-design process in our study, the content topics that were identified and co-developed offer unique insights into the SRH informational needs of immigrant adolescents in Canada. Participants emphasized that although puberty, menstruation, and bodily changes were basic SRH topics, they would appreciate culturally grounded discussions and insights into these topics (eg, tampons and myths). They also highlighted that immigrant adolescents do not always get the opportunity to learn about topics such as contraception or intimate relationships, and navigating access to confidential care, which is also supported by our previous studies and other literature [10,37]. Although platforms in Canada are increasingly adapting their approach to include culturally sensitive information, not many are designed specifically with immigrant adolescents in mind [13,16,17]. This further indicates the significance of this digital intervention.

While our process yielded valuable insights, it also presented challenges. Balancing adolescent preferences with evidence-based practices sometimes requires careful negotiation, which aligns with challenges noted in other co-design studies [20]. For example, when adolescents suggested including popular but scientifically unsupported remedies, we had to find ways to address these interests while maintaining medical accuracy. We navigated these situations by explaining the importance of scientific evidence, acknowledging the popularity of these remedies, and collaboratively finding ways to address the underlying concerns that made these remedies appealing. This approach ensured that adolescents felt heard and valued, even when their initial suggestions were not feasible. Unexpectedly, these negotiations led to innovative ways of communicating complex medical information in adolescent-friendly language, enhancing the overall accessibility of the content. Furthermore, to ensure the sustainability of adolescent involvement, we have established a rotating membership system for the AAG and are developing a mentorship program where experienced members can guide newcomers. This mentorship program will include training sessions for mentors, regular check-ins, and a 6-month mentorship cycle to balance continuity with fresh perspectives.

### Future Implications

Preliminary feedback on the intervention has been positive, with pilot users reporting high levels of engagement and improved SRH knowledge. Specifically, users praised the website’s discreet design, easy-to-understand language, and engaging features. However, long-term effectiveness studies are still ongoing, and findings from the usability testing are published elsewhere. These studies will track key metrics, including SRH knowledge retention, user satisfaction, frequency of website usage, and self-reported behavior change related to SRH practices. By monitoring these outcomes, we aim to comprehensively evaluate the intervention’s impact and identify areas for future improvement.

### Limitations

Through collaborative efforts in our co-design process, we actively and meaningfully engaged immigrant adolescents as active partners in developing the PEER digital SRH platform. However, our process also had its limitations. First, while our recruitment efforts aimed to include individuals from diverse gender identities and backgrounds, including LGBTQ+ youth and adolescents with disabilities, our advisory group lacked sufficient representation from these groups. Although this raises the question of the generalizability of our content to diverse groups, we ensured that our content was inclusive and included gender-neutral language. This was strongly emphasized by our AAG members and SRH experts working with adolescents. Second, our advisory group was predominantly female (n=23, 88%), and more than half of the participants were aged 18-19 years old. This demographic skew may limit the generalizability of our findings and could influence the content developed for the platform. The predominance of female voices may have emphasized certain gendered experiences and needs (eg, content on menstruation and pubertal changes), while perspectives of male and nonbinary youth may be underrepresented. To address this limitation, we intentionally explored the SRH information needs of male and gender-diverse adolescents by encouraging participants to discuss challenges faced by their peers across different gender identities. However, it can also be considered as a strength as participants were able to offer greater reflection based on their capacity to reflect on their previous experiences with SRH education. Our future research will aim to engage and seek a more gender-diverse group to evaluate the effectiveness of the intervention design and incorporate recommendations to reflect the full spectrum of adolescent SRH experiences and needs. Finally, maintaining consistent engagement over time and managing diverse opinions within the group required significant resources and facilitation skills from the research team. Future projects could benefit from establishing clear guidelines for resolving conflicts between adolescent preferences and evidence-based practices and developing long-term strategies for long-term engagement.

### Conclusion

Adolescents need access to accurate and reliable SRH information for positive sexual health outcomes and to prevent unintended consequences, such as STIs, unintended pregnancies, and so on. However, they do not always have the necessary means to secure such information. Digital interventions have the potential to influence health behaviors and promote positive outcomes by reaching a wide range of audiences, especially those who are at risk of being distanced from mainstream services. We used the principles of community-based participatory action research and HCD to co-design digital SRH interventions with adolescents by keeping their needs at the center of the process. By giving them a voice in the process and the ability to influence decision-making, we were able to co-develop a resource that has the potential to be user-friendly, accessible, effective, and sustainable among immigrant adolescents. This process allows the cocreation of culturally relevant interventions that have the potential to reach vulnerable populations, particularly in conservative societies where stigma and misinformation are pertinent.

## Data Availability

The datasets used and/or analyzed during this study are available from the corresponding author upon reasonable request.

## Abbreviations

AAG: adolescent advisory group
HCD: human-centered design
KT: knowledge translation
LGBTQ+: lesbian, gay, bisexual, transgender, queer or questioning, and plus (others)
PEER: Partnership to Empower Adolescent Engagement in Research
PI: principal investigator
SRH: sexual and reproductive health
STI: sexually transmitted infection
